# Impact of Ultrasonic Welding Parameters on Weldability and Sustainability of Solid Copper Wires with and without Varnish

**DOI:** 10.3390/ma17205033

**Published:** 2024-10-15

**Authors:** Andraž Logar, Damjan Klobčar, Uroš Trdan, Aleš Nagode, Gregor Černivec, Tomaž Vuherer

**Affiliations:** 1 Bosch Rexroth d.o.o., 4210 Brnik, Slovenia; andraz.logar@boschrexroth.si (A.L.); gregor.cernivec@boschrexroth.si (G.Č.); 2 Laboratory for Welding, University of Ljubljana, 1000 Ljubljana, Slovenia; 3 Department of Materials and Metallurgy, University of Ljubljana, 1000 Ljubljana, Slovenia; ales.nagode@ntf.uni-lj.si; 4 Laboratory for Welding, University of Maribor, 2000 Maribor, Slovenia; tomaz.vuherer@um.si

**Keywords:** solid copper wires, ultrasonic welding, varnish, joint strength, electrical conductivity, sustainability

## Abstract

This article contains an advanced analysis of the properties of solid wire electrical contacts produced by ultrasonic welding, both with and without varnish. The main disadvantage of ultrasonic welding of thin wires is the inability to achieve acceptable peel force and tensile strength, which is mainly due to the deformation and thinning of the wires. This study deals with ultrasonic welding using a ring of thin solid copper wires that minimises the deformation and thinning of the wires. The influence of welding parameters such as energy, pressure and amplitude were systematically analysed. Based on these parameters, the optimum welding programme and control method was determined to weld unvarnished and varnished wires. The investigations included electrical resistance tests, optical microscopy, micro-hardness measurements, peel tests and tensile tests, and the measurement of energy consumption. The results showed no significant differences in microstructure and hardness between varnished and unvarnished joints. Ultrasonic joints of varnished wires achieved lower electrical conductivity (by 38%), lower tensile strength (by 3%) and higher peel strength (by 7%), while the welding process was more sustainable in terms of energy (by 6.6%) and time consumption (without preprocessing).

## 1. Introduction

Electrical devices are playing an increasingly important role in our daily lives. In the production of high-performance electrical devices such as electric motors, battery connections, wiring and similar products, it is important that the connections have high electrical conductivity, high mechanical strength and high resistance to vibration, which increases the energy efficiency and service life of the product. The cabling consists of varnished wires, which must be removed before welding in the connection area. By eliminating the process of varnish removal, which consumes time, energy, resources and production space and generates dust, we speed up the production process and make it easier, faster and a little more sustainable. Each joining method has its own advantages and disadvantages that must be thoroughly evaluated to select the best technique for joining solid wires, conductors, or braids.

Common technologies for joining wires and conductors include crimping, soldering, electrical resistance welding and welding with mechanical energy [1]. Crimping, which includes both conventional mechanical and electromagnetic crimping [2,3,4], is a simple and cost-effective process. The strength of the connection is usually high due to the significant equilibrium pressure, which ensures the durability of the connection under various environmental conditions [5]. However, electrochemical problems can occur in the contact area of crimped connectors [6], resulting in higher electrical contact resistance, heating of the joints and energy loss [2].

Various heating techniques are used in soldering, such as oxyfuel, electrical resistance, induction and laser [7]. Soldering offers versatility and allows wires and conductors with different cross-sections and dissimilar materials to be joined without having to change the process parameters. The quality of a soldered joint depends on an optimal joint gap, sufficient overlap of the joint, the cleanliness of the joint, a homogeneous temperature throughout the joint and the stability of the influencing factors.

No reduction in the wire cross-section leads to a larger solder joint, which results in low electrical resistance and high joint strength. However, since the filler material and the base material have different chemical compositions, such joints are more susceptible to corrosion damage [8,9,10,11] and microstructural changes due to the heating process [8,12,13]. Takahashi et al. [14] reported that a high potential difference between the wire and the chemical composition of the filler material leads to a higher degree of deterioration. To improve wettability, the filler material often contains lead, which is toxic and harmful to the environment and human health [15]. In addition, silver solder, which is commonly used in copper soldering, is expensive and should be considered carefully as it is considered a critical raw material (CRM) [16].

Electrical Resistance Welding (ERW) utilises Joule heating to join materials, and several special techniques are available. These include (i) Hot crimping, which allows simultaneous removal of the varnish and welding of the wires; (ii) Compaction, where the connection is made by Joule heating in combination with deformation; (iii) Thermode process, in which a heated tungsten electrode first melts the varnish and then welds the wires together and (iv) resistance soldering, in which flux, solder and resistance heating are used for soldering [1]. These methods make it possible to join varnished wires but also have their limitations. They often require different electrodes and welding parameters for wires with different cross-sections, which limits their use in low-volume production.

Ultrasonic welding (UW) is an extremely promising way of welding with mechanical energy to connect wires and braids, as the varnish can be removed from the wires at the same time. An important advantage of ultrasonic welding is the possibility of welding a wide range of different cross-sections of wires and braids using the same tool and real-time quality control of the welding process. Stavropoulos et al. [17] report that ultrasonic welding creates joints with low electrical resistance, which enhances the overall efficiency of electrical connections. Welding of wires with small cross sections is a challenge due to the thinning of wires, but it can be successfully performed with a ring/collar [18]. Current scientific findings always address UW only in conjunction with pre-cleaned/unvarnished wires. Kuprys et al. [19] reported that a cleaner joint area produces a higher quality of the joint. Tsujino et al. [20] have explored the possibility of welding insulated cables and braids without removing the insulated jacket. A patent [21] has also been granted in this field. It is also known that with a ring/collar [21,22,23], we increase the peeling strength of cable bundles where the conductors of electrical wires are connected, even if at least one of the electrical wires has a small diameter. Zhou et al. [24] observed that prolonged welding times result in a reduction of the bonding area’s thickness due to deeper penetration by the sonotrode, subsequently diminishing the peak tensile strength of an Al/Ti joint. Similarly, Shakil et al. [25] found that increased energy levels enhance the sonotrode’s penetration depth, which compromises the mechanical strength of the joint. The sonotrode’s penetration alters and moves the material, making it thinner and softer, thereby adversely affecting the joint’s strength [26]. Dhara et al. [27] observed that increasing welding pressure also leads to a reduction in material thickness, which can transition the weld from under-weld to good-weld, but excessive pressure can also result in over-welding, reducing weld strength due to insufficient material flow and mixing. However, all that research was performed exclusively for welding or increasing the strength of the joint of conductors, braids, or multi-layered battery joints, where the welding area was cleaned or free of coatings. In industry, ultrasonic welding is mostly used with removed varnish, and limited knowledge is available in the scientific literature about the ultrasonic welding of lacquered, i.e., varnished solid wires, which presents a promising opportunity for new research.

The present study investigates the effects on connectivity and mechanical strength when ultrasonically welding three solid copper wires using a copper ring, both with and without varnish on the wires. The process of ultrasonic welding of metals is conceptually simple and involves three main variables: time, which measures the duration of the ultrasonic vibrations; amplitude, which describes the longitudinal displacement of the vibrations; and force, which denotes the compressive force exerted perpendicular to the direction of the vibrations. The multiplication of time, amplitude and force results in the energy used. Energy-based welding control is widely used in metal welding, especially when the welding surface is uncoated. However, alternative approaches may be required for coated surfaces, i.e., varnished wire surfaces. Based on the experimental research, we have developed a step-power process that enables the successful US welding of thin, varnished wires with the ring. A developed process consists of the melting of varnish with specified energy and higher amplitude and continues with the welding cycle at lower amplitude and energy. 

## 2. Materials and Methods

### 2.1. Materials and Preparation of Samples for Welding and Soldering

A 0.71 mm diameter wire made of C11000 copper coated with polyester and polyamide-imide coatings was used for experimental welding. Table 1 presents the properties of selected wires. The coating itself can withstand 200 °C [28]. The removal of varnish for selected samples was performed using Abiofix (Waldshut-Tiengen, Germany). 

**Figure 1:**
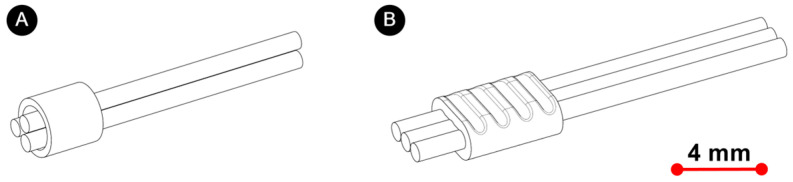
(**A**) A sketch of joint preparation for UW and (**B**) a sketch of the welded sample.

UW of thin wires is more successful if welding is performed with a ring. An inner diameter of 2 mm, an outer diameter of 3 mm and a length of 4 mm were the dimensions of the ring used. Figure 1 shows a schematic representation of the weld seam before (A) and after UW welding (B). 

### 2.2. Optimization of Ultrasonic Welding of Wires—The Influence of Control Method

**Figure 2:**
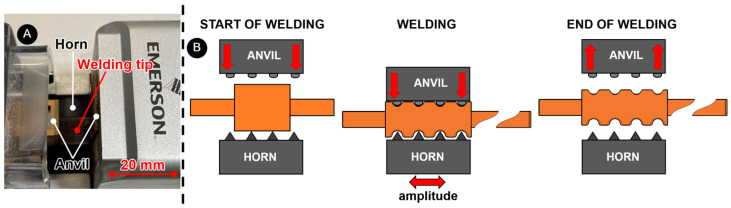
The GMX-W1 device with important parts is shown in (**A**), and the schematic describes the ultrasonic welding process in (**B**).

The experimental welding of the wires was performed by ultrasonic welding using the Branson GMX-W1 (Dietzenbach, Germany) ultrasonic wire splicer (Figure 2A). It converts electrical energy into mechanical vibrations of 20 kHz via a transducer. These vibrations are transmitted to the weld via the booster and a sonotrode (Figure 2B). A clamping of wires is crucial for successful welding, as well as proper welding parameters. 

A comparison of ultrasonic welding of copper wires with and without varnish was performed. Two different welding control methods were used for preliminary welding. The welding of the wires without the varnish was performed with the (i) ‘Welding To Energy’ control, while the welding of varnished wires was performed with the (ii) ‘Welding To Height’ control. In ‘Welding To Energy’ control, the most important triggering parameter for the formation of the joint is the energy used to form a joint. This energy is kept constant so that the time can be adapted to the condition of the materials during welding. ‘Welding To Energy’ control offers many advantages when welding materials with non-metallic oxides, grease and impurities on the surface. High-frequency cleaning, in combination with pressure force, cleans the weld seam partially at the beginning of welding [30]. The energy used is calculated using (Equation (1)).

(1) E=P·t

where *E* [J] is the energy, *P* [W] is the power and *t* [s] is the time. 

However, energy is no longer the most important parameter in welding varnished wires using ‘Welding To Height’ control. In UW of varnished wires, the height of the joint must also be considered. The varnished samples for the preliminary tests were welded using the ‘Welding To Height’ control, with the most important parameter being the final weld height. In our case, however, this method had the disadvantage that it was not repeatable, but the results provided information that was used in the development of the third welding control method. All varnished samples were welded using the (iii) ‘Step Power Welding’ control, where two amplitudes are used for welding. Here, the amplitude changes with the set power input. The energy that can be applied to the joint is also limited.

For all three welding methods, (i) ‘Welding To Energy’, (ii) ‘Welding To Height’ and (iii) ‘Step Power Welding’, all welding parameters were continuously adjusted with a closed loop control system. The closed loop monitored the precise setting of splice width and height, welding energy, welding force, amplitude, and power. Experimental welding parameters are shown in Table 2. 

### 2.3. Comparison and Testing of Varnished and Unvarnished Joints

#### 2.3.1. Measurement of Electrical Resistance 

**Figure 3:**
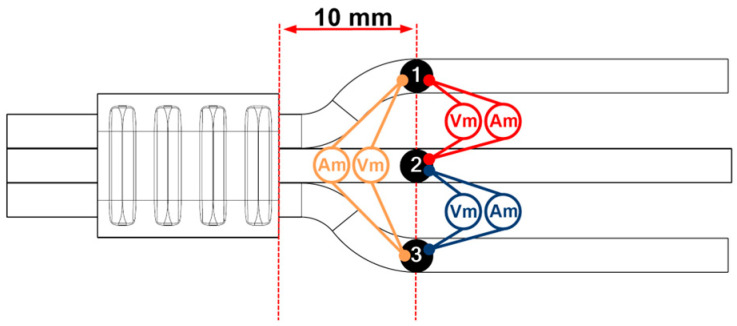
Display of contact layout for electrical resistance measurement. Numbers presents the measurement points.

Electrical resistance measurements were performed on 3 samples from each UW batch using the Keysight 34420A (Böblingen, Germany) nano-volt/micro-ohm metre. The 4-wire resistance measurement was used. Prior to testing, the wires were also ground at the attachment point of the metre. The electrical connection resistance was measured on all three wires. The connection between the individual wires was checked at the points shown in Figure 3. The connection terminals of the wires were 10 mm apart. Each sample was measured 3 times, and the average of the data was taken in order to obtain reproducibility of the results.

#### 2.3.2. Analysis of Macro/Microstructure and Microhardness Measurement

After welding, the joints were sectioned in the middle of the joint and embedded in epoxy resin. The specimens were ground (with 320, 500, 800, 1200 and 4000 grit abrasive paper), polished with a diamond paste DP-Suspension P and oxide polished with OP-S on the Struers device Abramin (Ballerup, Denmark). The prepared samples, i.e., the macrosections, were visually examined using the Keyence VHX-6000 (Neu-Isenburg, Germany) optical microscope (OM).

**Figure 4:**
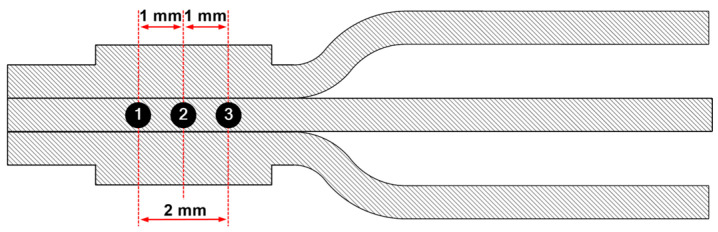
Display of the placement of the microhardness measuring points.

CT scan examination of the weld area of the samples was performed using the Nikon XT V 160 iCT (Tokyo, Japan) device. The microhardness was measured using the ZHU/ZwickiLine+ (Ulm, Germany) universal hardness testing machine, which measures the Vickers hardness HV0.2 according to the ISO 6507 standard [31]. The results were processed using the Zwick testXpert V12.3 hardness edition testing software. Hardness measurements were conducted in the same manner at least two times on both welded samples, i.e., with and without varnish on the surface. Afterwards, the average of the data was taken to obtain reproducibility of the results. Figure 4 shows the exact location of the measuring points. The hardness was only measured in the centre of the welding area, as it has already been proven that UW has no influence on the hardness outside the welding area [18].

#### 2.3.3. Testing of Mechanical Properties

**Figure 5:**
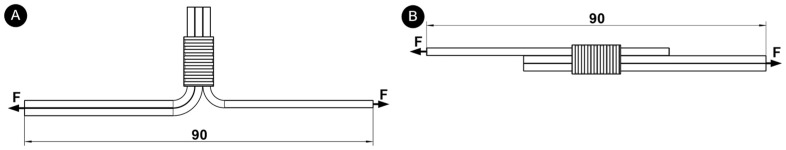
Mechanical test: peel force test (**A**), tensile strength test (**B**).

The tensile strength tests and peel force tests were performed in accordance with MAN Truck & Bus AG WORKS standard M 3455 [32]. The Zwick Z150 (Ulm, Germany) universal tensile testing machine with the KAP-S 2 kN load cell was used to perform both tests. The tests were performed using the Zwick TestExpert II V 3.5 software. Both tests were performed with the pulling speed of 50 (+5) mm/min. The specimens were clamped in grippers specially developed for testing wires and cables. The peel force test was performed on 10 UW specimens welded without varnish and 10 UW specimens welded with varnish (Figure 5A), while the tensile tests were performed on 10 UW unvarnished specimens, 10 UW varnished specimens and 10 wires (Figure 5B).

## 3. Results and Discussion

### 3.1. Preliminary Tests of Ultrasonic Welding of Unvarnished Wires Using ‘Welding to Energy’

**Figure 6:**
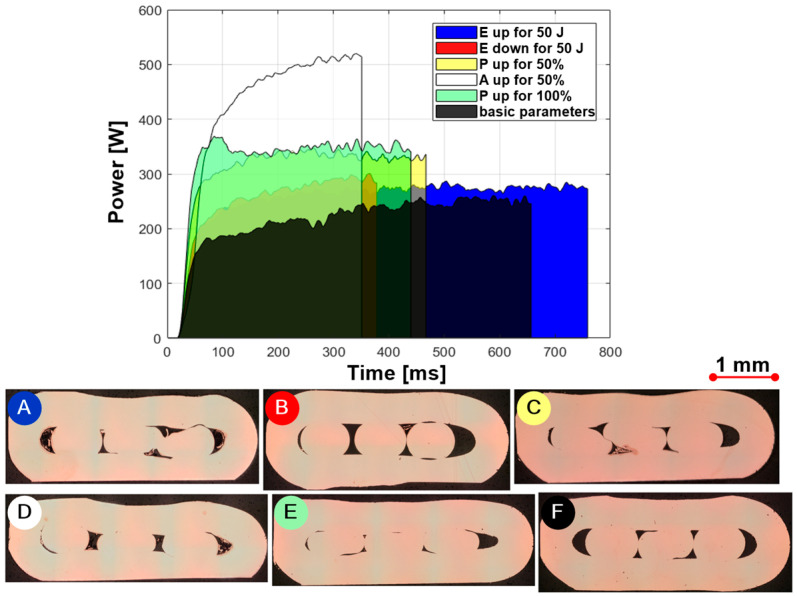
The influence of individual parameters on the ultrasonic welding process: 50 J more energy (**A**), 50 J less energy (**B**), 50% more pressure during welding (**C**), 50% bigger amplitude (**D**), 100% more pressure during welding (**E**) and basic parameters (**F**).

To better understand the influence of each ultrasonic welding parameter, a series of samples were welded using the ‘Welding To Energy’ method. Only one parameter was changed and analysed at the time: energy used (E), pressure (P) and amplitude (A). Five identical samples were made for each changed parameter. The welding device collected the parameters as a function of time and power, resulting in a surface representing the energy used for welding (Figure 6). As the energy [E] required to make a joint increases, the welding time increases, greater friction is achieved, and the weld becomes flatter (Figure 6A). If the energy is reduced, the welding time is shortened, the weld seam is higher since less friction generates less heat and smaller wire deformation and the weld strength between the wires and the ring is poorer (Figure 6B). Energy is a product of time and power. With the same welding power, the time increases with higher energy and vice versa with lower energy. If the pressure [P] increases (Figure 6C,E), the power must be increased to generate the same amount of energy. This, in turn, means a reduction in the welding time. The wires become more deformed. The test results also show that the amplitude [A] has a much greater influence on the power than the pressure. As the amplitude increases, the oscillation of the sonotrode increases, as does the mixing of the materials in the joint (Figure 6D), which requires more power. This means that welding is faster for the same energy input. This oscillation can also be used to remove the varnish from the wire at the beginning of welding. The parameters with which the individual samples were produced are described in Table 3.

If only the energy for producing the welded joint is considered, a repeatable and high-quality welded joint cannot be achieved due to a lack of sufficient friction due to the varnish. Consequently, the wires will simply break.

Based on the previous studies of unvarnished and varnished wires being ultrasonically welded, Mostafavi’s findings [33] and the SAE/USCAR-38 standard [34] preliminary welding tests were performed on 90 varnished samples. The ‘Welding To Height’ method was chosen to prevent wire breakage. The parameters used are described in Table 4.

### 3.2. Preliminary Tests of Ultrasonic Welding of Varnished Wires Using ‘Welding to Height’

**Figure 7:**
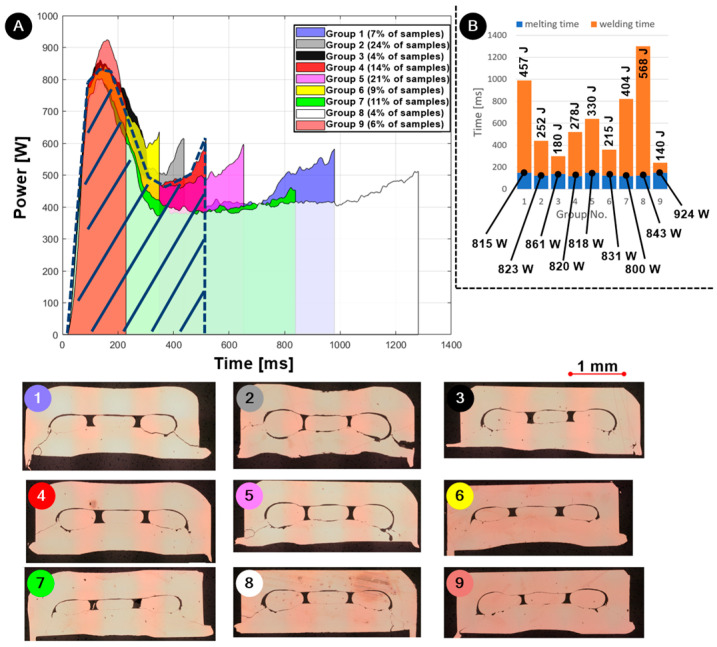
The influence of varnish on the ultrasonic welding process: power-time plot of each group (**A**) with their cross-sections; hatched area is desired power distribution for quality joint, (1–9) and column plot with average peak power (**B**).

Figure 7A shows the welding characteristics of varnished wires. The power-time plots differ between the welded samples. The reason for this is the coefficient of friction, which depends on the position of wires and thickness of varnish, heat dissipation, etc. Welding of 90 varnished wire samples produced power-time plots, which we divided into nine different group characteristics. Most samples (59%) of groups 2, 4 and 5 were welded in 520 ms ± 97 ms with a peak power of 820 W ± 28.8 W. It should be noted that 6% of samples were welded in 240 ms ± 10 ms with a peak power of 924 W ± 29.4 W. These were the samples where the friction coefficient was the highest. On the other hand, 4% of the samples from group 8 were welded in 1320 ms ± 63 ms with a peak power of 843 W ± 20.3 W. These samples had lower coefficients of friction, probably due to thicker varnish or the position of wires. 

It can be concluded from these results that the presence of varnish on the wires has a strong influence on the energy input into the joint itself and on the repeatability of the process. The instability of the process is also reflected in the range of energy introduced into the weld, which extends from 139 J to 645 J.

In the column plot in Figure 7B, the power required to melt the varnish at a high temperature is about 800 W for all groups. The plot also shows the energy required to form a joint for each group. The cross-sections in Figure 7A 1–9 show that the high amplitude required to remove the varnish at the same time produces cracks on the ring, reducing the weld joint strength. It can also be noted that the wires coated with varnish are welded to the ring and not to each other. This indicates that the ring itself not only increases the joint’s strength [18] but also enhances electrical contact. The ring protects the wires, as the deformations caused by the tool occur on the ring. The wires remain undamaged without reduced cross-section. The ring also increases the cross-section in the joint, which increases the electrical conductivity.

The impact of the cracked rings can also be confirmed with the analysis of the force-elongation curve both in the tensile test and in the peel force test. For example, the peel strength of group No. 4, with only small cracks present, proved to have the highest strength of 69 N ± 5.05 N. The peel strength of group No. 2 was among the lowest due to the presence of big cracks in the ring was 50.8 N ± 14.4 N. Tensile strength results correspond to the peel test results. The tensile strength of group No. 4 was 56 N ± 0.91 N, and the tensile strength of group No. 2 was 37.9 N ± 2.67 N. 

The upper part of the ring is fixedly attached to the anvil, while the lower part of the ring oscillates together with the sonotrode. Cracks occur due to the high amplitude. Cracks indicate that, at some point, the ring breaks into two parts. This results in the loss of additional support that protects the ultrasonic joint against peel forces. 

The electrical resistances between individual wires were measured for different welding group characteristics. For example, the electrical resistance of group No. 4 with the higher strength was 1589.5 µΩ ± 53.8 µΩ. Other results on peel force, tensile force and electrical resistance can be found in Table 5. If there were too few samples, the result was not recorded.

All results indicate that the highest weld strength and the lowest electrical resistance are obtained in group No. 4, where the welding time was 520 ms ± 26 ms and the energy required to form a joint was 278 J ± 12.9 J. From the welding characteristics in Figure 7A (hatched part), it is also seen that the power needed to start welding for group No. 4 was 820 W ± 32.7 W.

Based on these results, it was concluded that a large amplitude during the entire welding process/time damages the weld strength by producing cracks on the ringside close to sonotrode vibrations. Additionally, a high amplitude is needed, at least at the beginning, to quickly heat the joint to melt and remove the varnish. Based on these facts, the welding cycle was divided into two parts. 

To confirm the preliminary tests, the step-power welding method with limited energy input was selected. To reach the melting of the varnish, an energy of 800 W and an amplitude of 52 mm was selected. In the second part of the welding cycle, i.e., after using 800 W of energy, the amplitude drops to the welding amplitude of 20 mm. The selected parameters are listed in Table 6.

### 3.3. Welding of Varnished (‘Welding to Energy’) and Unvarnished (‘Step-Power Welding’) Wires with Prescribed Parameters

**Figure 8:**
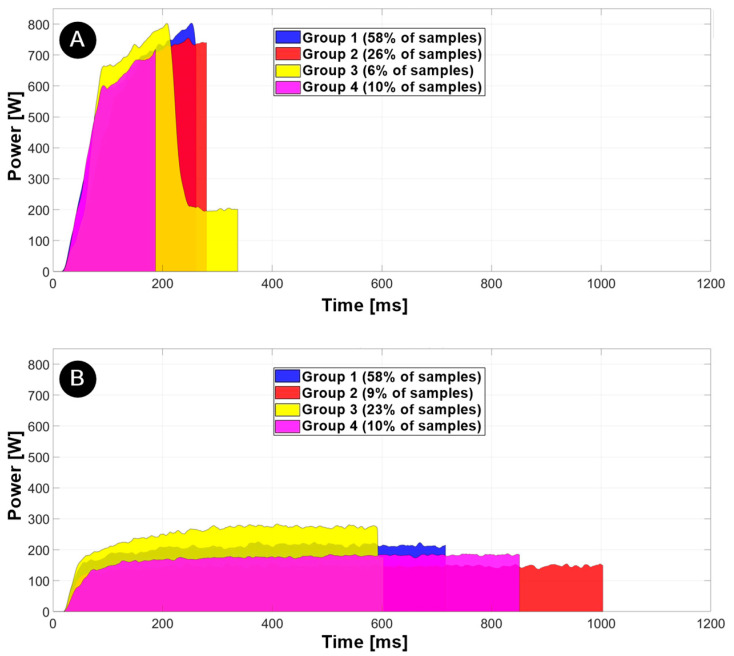
Power-time plot of varnished samples for each group; welded with step-power welding method (**A**) and power-time plot of unvarnished samples for each group; welded with welding to energy method (**B**); *n* = 30.

Thirty varnished samples were welded using the step-power welding method. As can be seen from the welding power-time diagram in Figure 8A, the diagrams differ between the welded samples due to the coefficient of friction, which depends on the position of the wires in the weld joint, the cleanliness of the surfaces, the heat dissipation, etc. We found that 10% of the samples were welded in 190 ms ± 22 ms with a peak power of about 715 W ± 11.8 W. In these joints, we did not apply 140 J of energy, but we still obtained a quality joint without cracks in the ring. We hypothesise that this is due to a thinner coating layer, which increases friction and, therefore, reduces the welding time. Most of the samples (84%) were welded in 260 ms ± 10 ms with a peak power of 734 W ± 45 W, while only 6% of the samples were welded in 340 ms ± 25 ms with a peak power of 800 W ± 4 W. Only in group 3, a decrease in amplitude can be observed when 800 W was reached at 220 ms ±. This decrease does not occur in most of the joints, as the maximum energy of 140 J is already introduced into the joint beforehand. However, it is clear from these examples that the power is reduced to the same level as when welding without varnish.

For comparison, 30 unvarnished samples were welded with a constant energy of 140 J. The diagram of the welding power in Figure 8B shows that the power-time curves also differ here. It was found that 23% of the samples were welded in 625 ms ± 22 ms with a peak power of 272 W ± 9.5 W. Most of the samples (58%) were welded in 700 ms ± 32 ms with a peak power of 248 W ± 13.6 W, while only 9% of the samples were welded in 1000 ms ± 40 ms and with a peak power of 157 W ± 9.1 W. These latter samples had some varnish residues on the wires, resulting in a lower coefficient of friction, lower welding stability and longer welding times when welding at constant energy. Similar conclusions were also made for less clean welds, which were also reported by Kuprvs et al. [18] in their study on the welding of copper wire.

### 3.4. Analysis of Varnished and Unvarnished Joints 

#### 3.4.1. Testing of Electrical Resistance 

**Figure 9:**
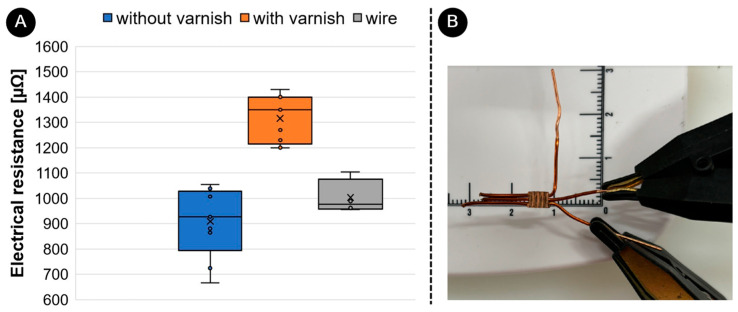
Electrical resistance measurements: (**A**) Comparison of electrical resistance between the UW joints without varnish (blue), UW joints with varnish (orange), and wire (grey) (*n* = 5) and (**B**) positions of measurement points on the example of UW joint without varnish.

To quantitatively evaluate electrical resistance between unvarnished and varnished UW joints, Figure 9 shows a box-whisker diagram comparing the electrical resistance between them. The measurements were carried out on 5 unvarnished and 5 varnished wires. Each sample was tested 3 times. The electrical resistance of unvarnished UW samples was 982 µΩ ± 161 µΩ. The electrical resistance of varnished UW samples was 1350 µΩ ± 86 µΩ. The electrical resistance of the wire itself was 976 µΩ ± 60 µΩ. The mean values show that the results are in a similar range. It is obvious that the electrical resistances of varnished joints are 38% higher than those of unvarnished joints due to the significant influence of the varnish in the joint area. It can also be observed that the electrical resistance values between the individual wires in the joint do not differ significantly between the welding methods used, which shows that a good joint is created between the ring and all three wires. A similar study was carried out with an ultrasonic (unvarnished) and a soldered joint, whereby it was found that the soldered joint had a 28% higher electrical resistance [18].

#### 3.4.2. Analysis of Macro/Microstructure, CT-Scan and Hardness 

**Figure 10:**
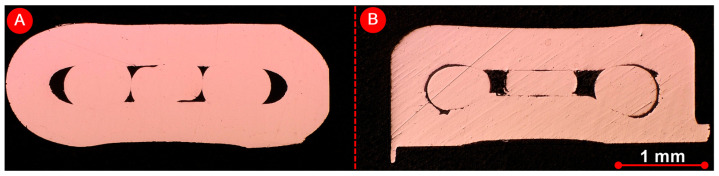
Analysis of the microstructure of the UW joint (wire and ring) (**A**) without and (**B**) with varnish.

Both the macrosection analysis and the microstructure analysis were used to carry out a detailed evaluation of the weld zones. Figure 10 shows that there is a strong metallurgical bond between the wire and the ring in both UW samples (Figure 10A) (unvarnished wires) and (Figure 10B) (varnished wires). In the joint of unvarnished wires, the connection between the wires and the ring is visible on the side of the sonotrode and on the opposite side of the sonotrode. In contrast, the connection between the ring and the varnished wires was only made on the side of the sonotrode. There is also a remarkable mixing of the wire and ring materials. The relative movement required to remove the varnish is most pronounced here.

**Figure 11:**
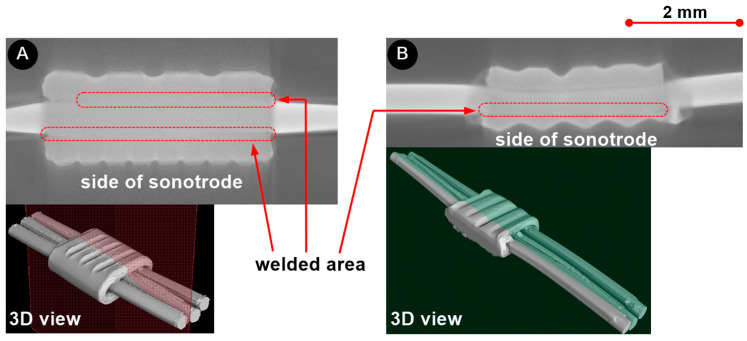
Analysis of CT scan of the UW joint (wire and ring) (**A**) without and (**B**) with varnish.

Figure 11 shows the CT scan of the unvarnished and varnished sample. The CT scan confirms the results of the cross-section analysis. The CT scan shows (Figure 11A) that when welding unvarnished wires, the joint is located on both the lower and upper sides of the ring, whereas, with varnished wires (Figure 11B), the joint is located only on the lower side of the ring, which is closer to the sonotrode. It can also be seen that the connection runs across the entire ring.

**Figure 12:**
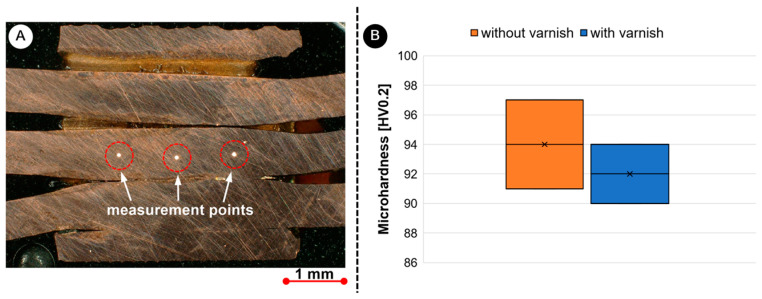
Microhardness HV0.2 measurements of UW joints: (**A**) positions of microhardness measurement points on the example of joint with varnish and (**B**) results of microhardness measurements.

Figure 12 shows the microhardness measurements taken at three specific points of both UW samples in the centre of the joint. The average hardness values for unvarnished UW joints were 94 HV0.2 ± 4.8 HV0.2, and for varnished UW joints, 90.5 HV0.2 ± 8.5 HV0.2. For the varnished joints, a slight decrease in hardness was observed (3.5 HV0.2), which is probably due to the softening effect [18]. Welding the varnished wires requires a higher energy input (800 W compared to 270 W) into the welding area, which also means a higher generated temperature in the welding area.

#### 3.4.3. Analysis of Mechanical Properties

**Figure 13:**
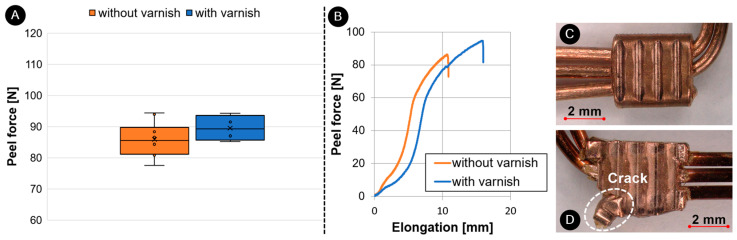
Comparison between properties of UW joints with and without varnish: (**A**) peel force, (**B**) peel-force–elongation curves, fracture of peel-force samples, (**C**) without varnish and (**D**) with varnish.

Figure 13 shows a comparison of the peel strength results of ultrasonic welded unvarnished and varnished wires. The peel strength of unvarnished joints was 85.6 N ± 5.2 N, and that of varnished joints was 91.5 N ± 3.8 N. Ultrasonic welding of varnished wires resulted in more robust joints with 7% higher peel strength. The force–elongation curve (Figure 13A) shows that the elongation values are higher for the UW specimens welded with varnish. The peel force–elongation curve (Figure 13B) also shows a drop in force at around 79 N for the varnish welded specimens. This can be attributed to the higher deformation of the ring during welding with a higher amplitude. Figure 13D shows that the ring cracks during the peel test at the varnished joint. This phenomenon was not observed in samples welded without varnish (Figure 13C). A similar study was carried out with an ultrasonic (unvarnished) and a soldered joint, whereby it was found that the soldered joint had a 42% lower peel strength [18].

**Figure 14:**
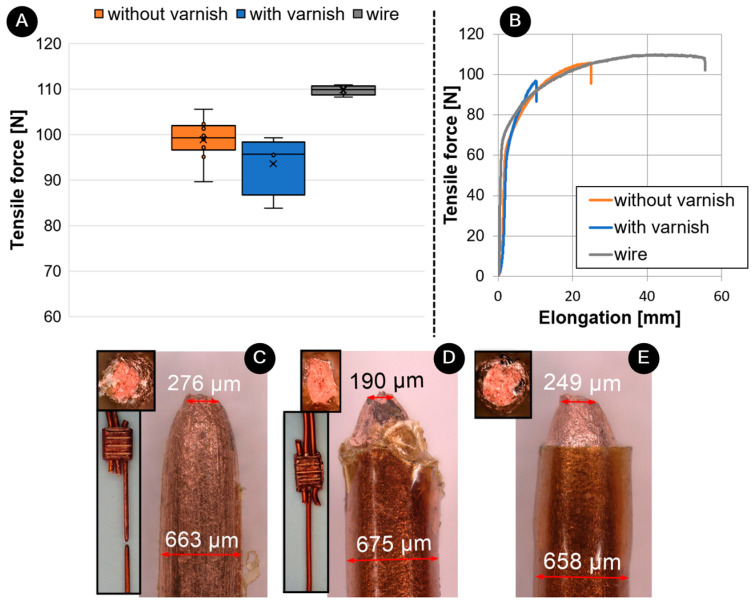
Comparison between tensile properties of wire and UW joints with and without varnish: (**A**) tensile force, (**B**) tensile-force–elongation curves, (**C**) fracture of unvarnished joint, (**D**) fracture of varnished joint and (**E**) fracture of wire.

Figure 14 shows the results of the tensile strength test. The tensile strength of unvarnished joints was 99.3 N ± 4.2 N, compared to varnished joints with 96.3 N ± 7.9 N. The tensile strength of the wire was 109.9 N ± 0.95 N. The UW joints produced on wires without varnish had a 3% higher strength (Figure 14A), which also corresponds with the fact that it breaks outside the weld seam, in contrast to the varnished joint, which breaks at the end of the weld (Figure 14C,D). Nevertheless, the tensile strength of the wire itself is 11% higher than the unvarnished joint because the varnished wire has additional strength in the coating/varnish itself. The force-elongation curve (Figure 14B) showed that the elongation of the UW joints without varnish was 50% smaller than that of the wire. The wire is deformed during UW, resulting in a more brittle fracture (as the cross-section reduces) [18]. It can also be seen that the elongation of the UW joints with varnish was also 50% smaller than the elongation of the UW joints without varnish. This can also be confirmed with the final height of the joint after welding. The height of the UW joint without varnish was 1.83 mm ± 0.13 mm, while the height of the varnished joint was 1.07 mm ± 0.05 mm. The deformation of the varnished joint is 42% higher than that of the unvarnished joint simply due to the final height. This can also be seen in Figure 14C–E, where the height of the varnished wire fracture cross-section is smaller than that of the wire itself or unvarnished wires. The wire itself was not subject to any deformation. Another reason is also the higher deformation during UW with higher amplitude to achieve the varnished wire joints. A similar study was carried out with an ultrasonic (unvarnished) and a soldered joint, whereby it was found that the soldered joint had a 12% lower tensile strength [18].

### 3.5. Explanation of Joining and Failure Mechanisms

**Figure 15:**
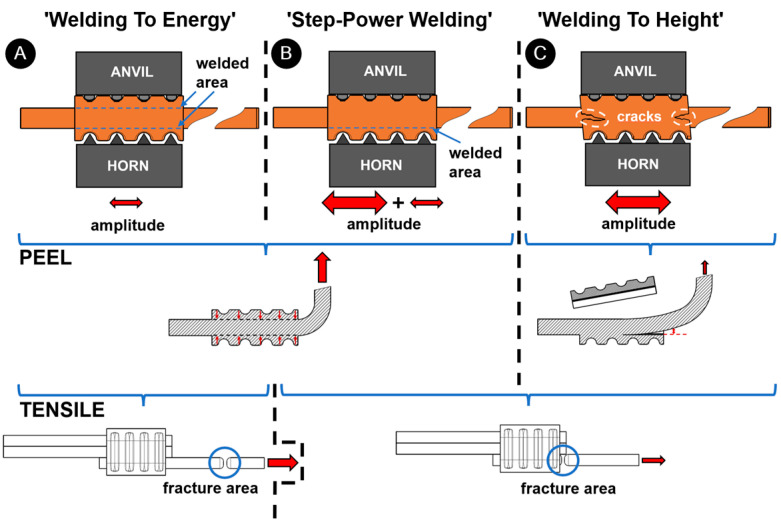
The mechanism of joining and failures are as follows: (**A**) Welding to energy, (**B)** Step-Power Welding, and (**C**) Welding To Height control. The size of two-sided arrows presents the size of the amplitude. One-sided arrows presents the direction of force during the mechanical testing. Blue circles present the breaking point of the joint.

The mechanism of joint formation (Figure 15) varies slightly between the processes. When welding unvarnished wires, the most important parameter for successful joining is the energy. Since there is no varnish on the wire surface, a lower amplitude is sufficient to achieve sufficient friction and, thus, a high-quality joint. Although the ring is fixed on the upper side and vibrations come only from the lower side, this vibration is too small to damage the ring (Figure 15A). In contrast, when welding varnished wires, a high amplitude is required to heat, melt and remove the varnish to clean the joint area. This high amplitude has a negative effect on the ring as the oscillation causes cracks in the ring (Figure 15C). To weld varnished/enamelled wires, a high amplitude must only be used at the beginning to remove the varnish, but later, a lower amplitude is sufficient for the welding (Figure 15B). In this way, a joint is achieved without damaging the ring. Such joint has good metallurgical bonding between the wires and rings close to the sonotrode, while limited bonding is performed on the anvil side of the ring; for unvarnished samples, good metallurgical joint is obtained on the anvil and sontrode side.

The energy consumption to produce an unvarnished or varnished joint during the welding process was the same since, in both processes, the welding was controlled by specified energy. The actual power consumption was also checked with the Metrel Power Q4 (Horjul, Slovenia) network diagnostic tool. Here, energy consumption and losses were measured using 10 welding samples for each process. The energy consumption for welding unvarnished wires was 1484.4 J ± 120.79 J, and for varnished wires, it was 1385.3 J ± 151 J. As shown, less energy is consumed when welding varnished wire because the desired energy is consumed in a shorter time since different welding parameters (i.e., high amplitude) were used. Our unpublished study on energy consumption showed that soldering consumed 20,482.5 J ± 1590.28 J energy for production of the same joint, which is 13 to 15 times higher than ultrasonic welding. 

Ultrasonic welding of coated wires without removing the varnish is not commonly used in demanding applications for component joining. Nevertheless, this method offers several advantages, such as eliminating the need for insulation removal, reducing dust generation in the production environment, and decreasing both space and energy consumption. From an economic perspective, considering time, ultrasonic welding without varnish is 2.4 times slower than welding with varnish. The scientific contribution and research lie in the division of process control into two steps. Based on experimental work, we developed a welding method consisting of two stages: first, with high amplitude, followed by low amplitude. However, this method does not result in welding on the side of the support tool (ANVIL), and with inappropriate parameters, ring tearing occurs. Despite these challenges, this welding method enables the creation of weld joints that are almost comparable in quality to those made with insulation removal. 

## 4. Conclusions

This study compares the ultrasonic welding of unvarnished and varnished thin copper wires with a ring. Here, different process control programs and parameters were used to obtain optimal joints. The comprehensive evaluation includes preliminary tests, welding parameters analyses, electrical resistance analyses, microstructure analyses and peel and tensile strength tests. The main conclusions are summarised as follows:

Welding to energy is an optimal controlled process for welding unvarnished wires, where 180 J of energy is optimal for welding selected joining combinations. 

Welding To Height: A higher sonotrode amplitude significantly enhances welding peak power by improving material mixing and reducing welding time. In ultrasonic welding of varnished wires, the varnish affects energy input and process repeatability, leading to considerable variations in energy introduced into the weld seam when using the suggested welding-to-height method. High amplitude is required to achieve the necessary power (800 W) and temperature to melt the varnish, but this causes ring cracks, which reduce the joint strength.

Two-Step Welding Process: A two-step welding process with varying amplitudes improves joint strength. Initially, a high amplitude of 52 µm is used to achieve 800 W power to heat the material and remove the varnish. Subsequently, a lower amplitude of 20 µm is applied to prevent ring cracking during welding.

Electrical Conductivity: The electrical conductivity of unvarnished ultrasonic welding (UW) joints is 38% higher than that of varnished joints. Unvarnished joints show a good metallurgical bond on both the sonotrode and anvil sides, whereas varnished joints have a good bond only on the sonotrode side. The reason for that is the changed welding parameters in the two-step welding process control, where sonotrode amplitude was reduced in the second step to avoid ring cracking. 

Microhardness: Microhardness values were 4% lower for varnished joints (90.5 HV vs. 94 HV) since the higher power is needed to heat and melt the varnish, which softens the wires. 

Mechanical Strength: Mechanical tests indicate that joints on wires with varnish have a 3% lower tensile strength (96.3 N vs. 99.3 N) and 7% higher peel strength (91.5 N vs. 85.6 N) due to ring deformation under force.

Energy Consumption and Sustainability: Ultrasonic welding of varnished wires is more energy-efficient, but the resulting joints have lower electrical conductivity compared to those of unvarnished wires.

The knowledge gained from this study provides a comprehensive understanding of the effects of varnish on the wires during ultrasonic welding. Based on the results, we conclude that welding of varnished wires produces high-quality joints with stable and repeatable processes. However, the electrical properties of such joints are inferior compared to those without varnish since some varnish remains between the ring and the wires. Despite this, the developed methodology can be effectively applied in industrial applications, particularly in the electrification and automation fields. An additional advantage of this method is that it eliminates the need for mechanical varnish removal, saving both time and energy and thus being more sustainable. On the other hand, this research demonstrates the impact of individual parameters on the ultrasonic welding process, which could serve as a solid foundation for further studies in the field of ultrasonic metal welding. To achieve the purpose of technical optimisation, the Design of Experiments (DoE) should be used with Response Surface Methodology, Orthogonal Design Methodology or other methods. Additionally, there is significant potential for improving our results in the welding of varnished wires, particularly in achieving a joint on the opposite side of the sonotrode. This will be the focus of our next research in this area.

## Figures and Tables

**Table 1:** The typical properties of enamelled round copper wire are 0.500 mm, with insulation film grade 1 according to standard DIN EN 60317-13 [28,29].

Properties	SHTherm^®^ 210
*Temperature index* [°C]	210
*Scrape resistance* [N]	≥7.500
*Elongation at break* [%]	≥38
*Electrical conductivity* [m/Ωmm^2^]	58–59

**Table 2:** Welding parameters and process control type for all three methods.

Parameters	‘Welding to Energy’	‘Welding to Height’	‘Step-Power Welding’
*Energy—E* [J]	140	/	140
*Pressure before and during welding—P* [bar]	1.38	1.14	1.38
*Amplitude—A* [µm]	20	52	52/20 *
*Jaw width—JW* [mm]	4.46	3.4	3.4
*Joint height—JH* [mm]	/	1.6	/

* Amplitude changes at a power level of 800 W.

**Table 3:** Plan of experimental parametric analysis of ultrasonic welding of unvarnished wires.

Parameters	BASIC	E↑ (50 J)	E↓ (50 J)	P↑ (50%)	A↑ (50%)	P↑ (100%)
*Energy—E* [J]	140	**190**	**90**	140	140	140
*Pressure before/during welding—P* [bar]	1.38	1.38	1.38	**2.07**	1.38	**2.76**
*Amplitude—A* [µm]	20	20	20	20	**30**	30
*Jaw width—JW* [mm]	4.46	4.46	4.46	4.46	4.46	4.46

**Table 4:** Welding parameters–preliminary tests on varnished wires.

Parameters	‘Welding to Height’
*Pressure before and during welding—*[bar]	1.14
*Amplitude—A* [µm]	52
*Jaw width—JW* [mm]	3.4
*Joint height—JH* [mm]	1.6

**Table 5:** Peel force, tensile force and electrical resistance for all groups.

	*Peel Force* [N]		*Tensile Force* [N]		*El. Resistance* [Ω]	
*Group 1*	50.7	/	36.4	/	1974	±3267.47
*Group 2*	50.55	±12.50	37.9	±2.67	2069	±99.84
*Group 3*	63.8	/	/	/	/	/
*Group 4*	69	±5.05	57.4	/	1390	±53.80
*Group 5*	50.3	±5.43	47.5	±4.62	2670.1	±383.64
*Group 6*	52.7	/	40	±0.5	1273	±116.62
*Group 7*	47.6	±13.78	50.7	/	2073	±241.79
*Group 8*	58.8	/	/	/	2082	±399.52
*Group 9*	/	/	51.7	±9.8	/	/

The data was not obtained since there were less than three repetitions for the selected group.

**Table 6:** Welding parameters for ultrasonic welding with ring for three varnished wires with a diameter of 0.71 mm.

Parameters	‘Step-Power Welding’
*Energy—E* [J]	140
*Pressure before and during welding—P* [bar]	1.38
*Amplitude—A* [µm]	52/20 *
*Jaw width—JW* [mm]	3.4

* Amplitude changes at a power level of 800 W.

## Data Availability

The original contributions presented in the study are included in the article, further inquiries can be directed to the corresponding author.
